# Riverbed depth-specific microplastics distribution and potential use as process marker

**DOI:** 10.1007/s11356-024-34094-z

**Published:** 2024-07-04

**Authors:** Marco Pittroff, Constantin Loui, Sascha E. Oswald, Mathias Bochow, Jan Kamp, Georg Dierkes, Hermann-Josef Lensing, Matthias Munz

**Affiliations:** 1 Department Geotechnical Engineering, Federal Waterways Engineering and Research Institute (BAW), Kußmaulstraße 17, 76187 Karlsruhe, Germany; 2 Institute of Environmental Science and Geography, University of Potsdam, Karl-Liebknecht-Str. 24-25, 14476 Potsdam, Germany; 3 German Federal Institute of Hydrology, Am Mainzer Tor 1, 56068 Koblenz, Germany; 4 Helmholtz Centre Potsdam, GFZ German Research Centre for Geosciences, Section 1.4 Remote Sensing, Telegrafenberg, 14473 Potsdam, Germany

**Keywords:** Microplastics, Vertical distribution, Sediment, Freeze core, Sediment dynamics, Tracer, NIR imaging

## Abstract

Riverbed sediments have been identified as temporary and long-term accumulation sites for microplastic particles (MPs), but the relocation and retention mechanisms in riverbeds still need to be better understood. In this study, we investigated the depth-specific occurrence and distribution (abundance, type, and size) of MPs in river sediments down to a depth of 100 cm, which had not been previously investigated in riverbeds. In four sediment freeze cores taken for the Main River (Germany), MPs (≥ 100 µm) were detected using two complementary analytical approaches (spectroscopy and thermoanalytical) over the entire depth with an average of 21.7 ± 21.4 MP/kg or 31.5 ± 28.0 mg/kg. Three vertical trends for MP abundance could be derived, fairly constant in top layers (0–‍30 cm), a decrease in middle layers (30–60 cm), and a strong increase in deep layers (60–100 cm). The dominant polymer types were polyethylene (PE), polypropylene (PP), and polystyrene (PS). Polyethylene terephthalate (PET) and PP were also found in deep layers, albeit with the youngest age of earliest possible occurrence (EPO age of 1973 and 1954). The fraction of smaller-sized MPs (100–500 µm) increased with depth in shallow layers, but the largest MPs (> 1 mm) were detected in deep layers. Based on these findings, we elucidate the relationship between the depth-specific MP distribution and the prevailing processes of MP retention and sediment dynamics in the riverbed. We propose some implications and offer an initial conceptual approach, suggesting the use of microplastics as a potential environmental process tracer for driving riverbed sediment dynamics.

**Supplementary Information:**

The online version contains supplementary material available at 10.1007/s11356-024-34094-z.

## Introduction

Microplastic particles (MPs) are a modern emerging pollutant of anthropogenic origin with a ubiquitous global presence and high persistence in the environment (Sarijan et al. 2021; Kumar et al. 2021a; Bergmann et al. 2019; Auta et al. 2017, Pinto da Costa et al. 2019). Several studies have already identified rivers as major transport pathways for land-based MPs into lakes and oceans (Meijer et al. 2021). However, riverbank and riverbed sediments can also act as a temporary sink or long-term retention site for MPs (Drummond et al. 2022; Margenat et al. 2021). The particle retention and transport in fluvial systems is determined by a variety of physical and hydrological mechanisms like granular filtration, advective flow, hyporheic exchange, or retention in hyporheic biofilms (Kumar et al. 2021b; Waldschläger and Schüttrumpf 2020). The superposition of these processes, combined with the high variability in polymer characteristics such as size, density, shape, and degree of alteration, can result in high spatial (horizontal as well as vertical) variability of MP distribution in riverbed sediments (Waldschläger et al. 2022; Kumar et al. 2021b).

Until now, several studies have investigated the presence of microplastics in surface waters, while less research has focused on the accumulation potential of MPs in riverine sediments (Range et al. 2023; Bellasi et al. 2020; Kumar et al. 2021a). Recent studies found a high MP abundance in sediments of different fluvial systems worldwide and showed that riverbed sediments can be a significant accumulation zone in rivers (Sarijan et al. 2021; Kumar et al. 2021a). However, in most riverine sediment studies, only the top layer of 0–30 cm depth was sampled (Waldschläger et al. 2020; Prata et al. 2019; Kumar et al. 2021a). Some studies also examined vertical microplastic profiles in riverbed sediments down to 60 cm (e.g., Frei et al. 2019) and in other environments of the river system, such as floodplains, estuaries, or mangroves down to 100 cm (Yuan et al. 2023; Weber et al. 2022). Within the riverbed, MP abundance has been found to decrease (Bao et al. 2023; Zhou et al. 2021) or increase (Niu et al. 2021; Fan et al. 2019) with increasing depth, but a mechanistic interpretation of the processes driving MP accumulation or translocation in the riverbed is lacking.

Column experiments have been performed to better understand the processes of MP retention (Munz et al. 2024; Lim et al. 2023; Li et al. 2024) and fractionation (Tumwet et al. 2022) in saturated sediments, indicating that the MP retention efficiency increases with increasing MP particle size and decreasing sediment size. However, typically just uniform porous media or spherical plastic particles were used in laboratory columns, and physical and chemical influences on the particle transport were identified (Waldschläger and Schüttrumpf 2020; Okutan et al. 2022; Lim et al. 2023). These experimental considerations are not easily transferable to realistic field conditions with heterogeneous sediments, dynamic boundary conditions, and sediment relocation events. To date, there is still a need for a detailed field investigation of the distribution and transport behavior of MPs in riverbed sediments, and information on MP abundance at depths > 60 cm in riverbeds is totally unknown. However, this knowledge is important when aiming to clarify the environmental fate and accumulation potential of MPs in fluvial systems and can potentially be used to derive accumulation processes within the last decades (Weber and Lechthaler 2021; Weber et al. 2022).

Some studies have proposed using MPs located in deposited lake, marine, or floodplain sediments as a time marker for the “Anthropocene” epoch (from 1950) or using the vertical MP distribution as a chronological or stratigraphic marker dating recent sedimentary deposits (Zalasiewicz et al. 2016; Weber and Lechthaler 2021; Weber et al. 2022). In contrast to lake systems, fluvial systems are highly dynamic environments potentially disturbed by factors like variable sedimentation rate, remobilization and relocation of particles, disturbance, and erosion. These factors are mainly caused by fluctuating energy levels (e.g., storms or strong currents), such that historical trends in the sediment sequence may be indiscernible (Martin et al. 2022). We therefore propose that in these hydraulically high-energy systems, the depth-specific MP concentration patterns may be used as a process marker for sediment dynamics and potentially lead to a better understanding of the driving sediment deposition and hydro-morphological processes.

Two analytical approaches to identify and quantify microplastics have mainly been used so far, based on either spectroscopic methods, such as Fourier-transform infrared (FTIR) and Raman spectroscopy (Raman), or thermoanalytical methods (TAM), such as pyrolysis gas chromatography-mass spectrometry (pyr-GC/MS) and thermo-extraction desorption GC/MS (Ted-GC/MS) (Primpke et al. 2020). Both approaches can be used complementarily, since they detect either particle number and size or polymer mass. Microplastics analysis is time-consuming and intensive in processing, when particles < 100 µm are analyzed using spectroscopic methods such as FTIR (11 µm) (Liu et al. 2023; Cabernard et al. 2018) or Raman (5–10 µm) (Pittroff et al. 2021; Cabernard et al. 2018). A novel alternative in line with the spectroscopy approach is near-infrared (NIR) imaging spectroscopy, which enables researchers to investigate larger filter areas in a short time, although with a limited detectable particle size of currently 50–‍100 µm (Munz et al. 2023; Fiore et al. 2022). Other promising tomography approaches are under development to enable the detection of MPs in undisturbed sediment samples for more accurate process investigations in the future (Tötzke et al. 2024).

In this study, we examined vertical concentration profiles of MPs in river sediments along the Main River (Germany), particularly at riverbed depths of 100 cm, which had not been previously investigated. We used the freeze core technique to get an undisturbed sample structure down to deep sediments and analyzed the MP concentration in depth-segments of 10 cm with a unique combination of two complementary analytical approaches (NIR and TAM). The results provide detailed insights into the depth-specific occurrence and distribution (abundance, type, and size) of MPs in deep-layer fluvial sediments. Based on the depth-specific MP distribution, we elucidate the potential of microplastics to be used as a process marker to infer the driving processes behind sediment dynamics and hydro-morphological processes in the riverbed over the past decades. We summarize these implications, offering an initial conceptual approach that has the potential to be transferred to other river sections and sediment structures. Finally, we discuss the information required for its application and the limitations of the proposed approach.

## Material and methods

### Study site

**Fig. 1:**
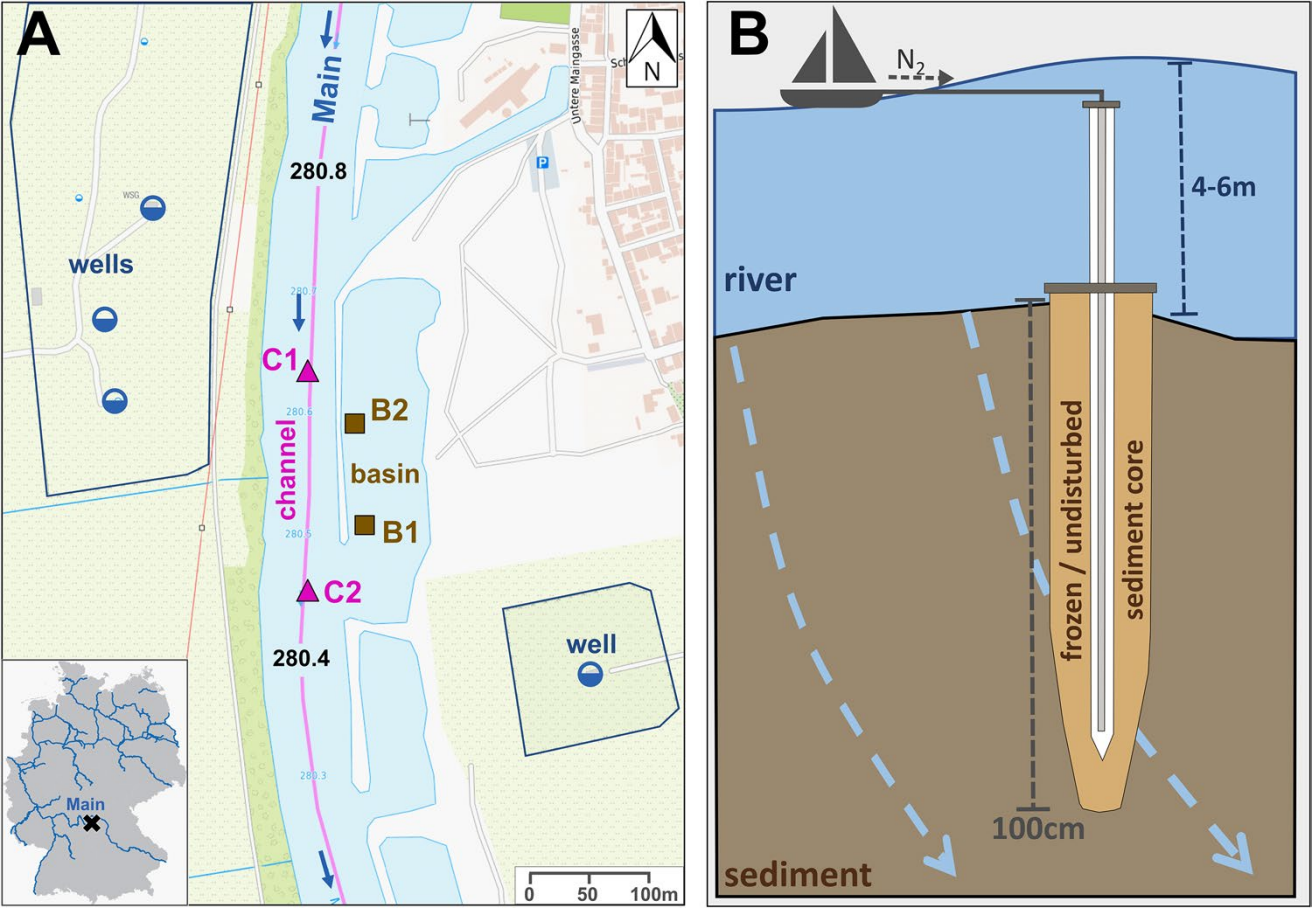
**A** Overview of the study site at the Main River (280.5 river-km) and nearby drinking water wells (half-filled circle icons). Location of the four freeze core sampling points; C1 and C2 at the shipping channel (triangle icons), and B1 and B2 at the river basin (square icons) protected by longitudinal training walls. Data source for the map: TopPlusOpen (©Federal Agency for Cartography and Geodesy). **B** Principle of freeze core sampling of undisturbed riverbed sediments at great water depths and deep sediment

The Main River (Germany) is the longest tributary of the Rhine River and flows through areas with substantial industrial influence and areas of high population density. Four sediment freeze cores up to a depth of 100 cm were collected from the riverbed close to a series of lateral basins at Marktsteft (Bavaria; 280.4–280.7 river-km), upstream of the Marktbreit barrage (275.6 river-km). Two cores (C1 and C2) were taken from the shipping channel and two (B1 and B2) from the river basin protected by longitudinal training walls (Fig. 1A). The study site was chosen because it provides permanently constant and well-known hydrogeological boundary conditions and a controlled hydrological situation, as described below. An important federal waterway in Germany, the Main River, is highly regulated. At the study site, the construction of the up- and downstream barrages was completed in the mid-1950s and last main waterway construction works of the riverbed and riverbank were finished in the early 1990s. Since then, no significant structure-altering construction works have been documented and no periodic maintenance works take place. Mean discharge (MQ) was 117 m^3^/s, mean flow velocity was 0.45 m/s, and water depths were between 4 and 6 m. Due to regulation by weirs, the water level shows only minor fluctuations of ± 10 cm. Water pumping (~ 260,000 m^3^/month) from nearby drinking water wells (100–160 m from the river) generates a groundwater drawdown and causes a continuous bank infiltration (~ 36% bank filtrate in wells) of river water (permanent losing conditions) into the quaternary aquifer (details in SI section S1.1). The groundwater flow rate to the wells was measured at approximately 1–‍2 m/day and hyporheic residence times were estimated to be ~ 80–‍400 h.

### Freeze core sampling

**Fig. 2:**
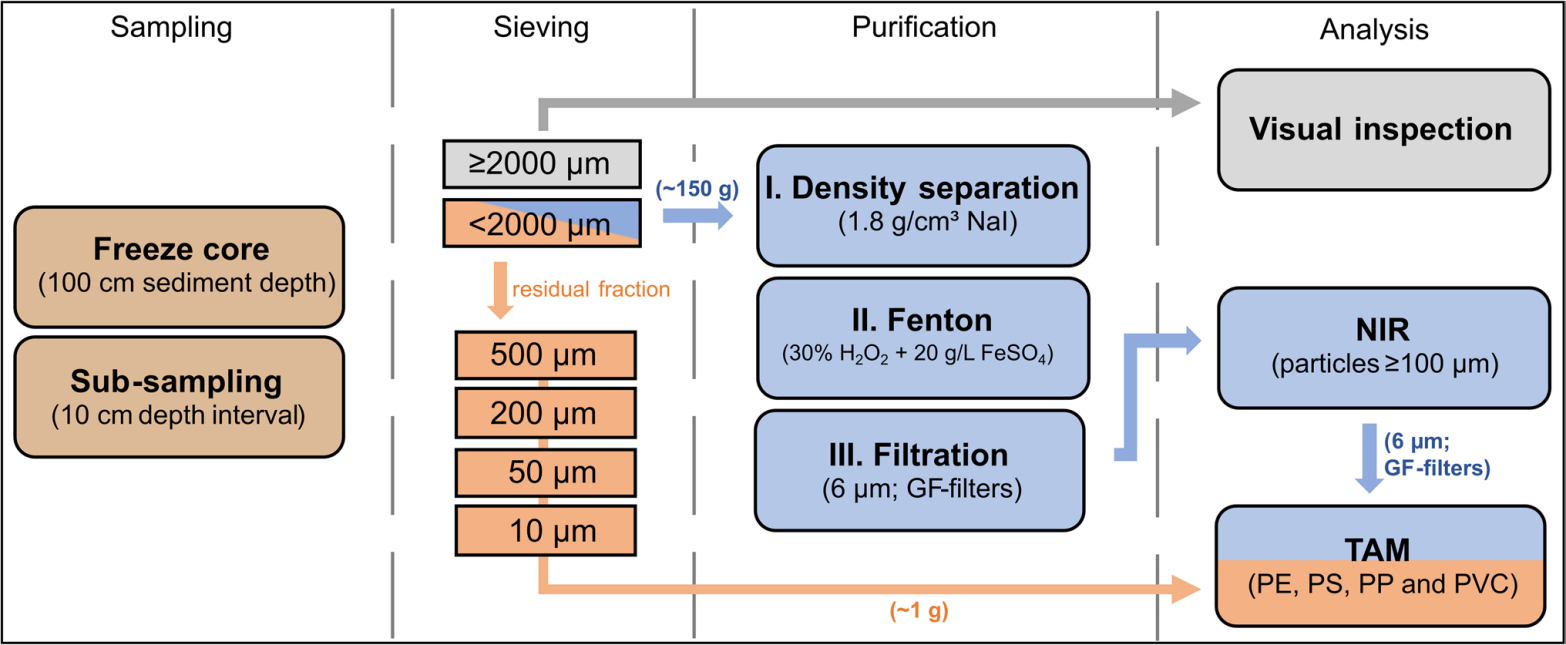
Overview of the overall analytical workflow from sampling to analysis using near-infrared (NIR) imaging spectroscopy and thermoanalytical methods (TAM)

The freeze core technique was used to get undisturbed, depth-oriented, and water-saturated sediment cores of 100 cm in length and 30–50 cm in diameter. The sampling method is particularly suitable in lowland rivers with relatively great water depths (> 2 m) and high flow velocities. A hollow metal lance was inserted into the riverbed and liquid nitrogen flowed through for 30–45 min, freezing the surrounding sediment and forming a solid core. For more details, see Strasser et al. (2015) and SI section S1.3. After sampling, freeze cores were transported frozen to the laboratory, wrapped in aluminum foil, embedded in sand for thawing, and sliced into 10 cm depth-segments. The sediment was oven-dried at 60 °C and sieved to a < 2 mm fine sediment fraction (stainless-steel sieves; Retsch GmbH, Germany) for further processing. The ≥ 2 mm coarse sediment fraction was visually inspected for plastics (no MPs found) and not processed further. The corresponding total dry weights of all depth-segments are listed in SI Table S6. The overall analytical workflow is shown in Fig. 2.

In C1 (0–‍50 cm) and C2 (0–‍45 cm), not enough fine sediment could be extracted by sieving due to the high-volume fraction of gravels and armourstones (62–71%) (Fig. 5). In B2 (60–100 cm), sample preparation (“Near-infrared imaging spectroscopy” section) was not sufficient to remove the high content of organic matrix (~ 107 g/kg) and the samples could not be analyzed further. In aliquots of sub-samples, the sediment grain-size distribution (ISO 17892–4 2016) and sediment textural classification (ISO 14688–1 2017) were determined for each depth layer. The organic matter content was estimated using the loss on ignition (heated at 550 °C for 4–8 h) according to DIN 18128 (2002). The hydraulic conductivity (*k*_f_) was estimated based on the grain size distribution (*d*_10_, diameter of the 10th percentile) and an empirically derived coefficient, as formulated by Hazen (1892). If the sample amount was insufficient for analysis, multiple depth-segments of the same sediment texture were combined.

### Near-infrared imaging spectroscopy

Approximately 150 g of fine sediment in each depth-segment was processed as follows (Fig. 2). First, density separation was performed using a high-density sodium iodide solution (NaI; ρ = 1.8 g/cm^3^) to isolate even high-density MPs from inorganics (Quinn et al. 2017). The sediment was placed in 250 ml wide-neck Erlenmeyer flasks, filled with the density solution, stirred and treated in an ultrasonic bath to destroy soil agglomerates, and left for 24 h. Subsequently, the supernatant was vacuum filtrated (6 μm stainless-steel mesh, Ø 47 mm; Sartorius, Germany). Second, the organic matrix was removed by applying a Fenton protocol treatment (30% H_2_O_2_ + 20 g/L FeSO_4_) as described by Al-Azzawi et al. (2020). This protocol was based on a stepwise addition of H_2_O_2_ at low temperatures (max. 60 °C longtime) with short reaction times (< 1 h). The sample volume was reduced to 4 ml by evaporation at 60 °C and a scaling factor of *K* = 20 ml was used to keep the ratio of reagent to sample constant. After reaction, 2–3 ml of 20% HCl was added to dissolve iron oxide precipitation; the sample was vacuum filtrated (10 μm stainless-steel mesh, Ø 47 mm) and subsequently transferred onto binder-free glass fiber (GF) filters (0.7 µm, Ø 47 mm; ROTILABO Typ CR263, Carl Roth, Germany).

The semi-automated MP identification approach of applying NIR imaging spectroscopy is described in full detail in Munz et al. (2023). In brief, a hyperspectral HySpex SWIR-320 m-e camera (Norsk Elektro Optik AS, Norway) with a wavelength range of 968–2498 nm, 256 spectral bands, and a pixel size of 50 µm coupled to a translation stage for scanning five filters in a row was used. The minimum identifiable MP diameter was 100 µm, since single pixel blobs were discarded to increase accuracy. Polymer identification of all detected MPs was done by automatically matching spectral patterns of each pixel spectrum against a spectral library containing the most common polymer types (listed in SI section S1.4) (Munz et al. 2023; Schmidt et al. 2018). All matched pixel spectra were visually cross-checked using the ENVI image analysis software (L3Harris Geospatial, USA) and particle diameter was calculated using the Python image processing tool (van der Walt et al. 2014). Uncertain matched particles were visually checked under a stereo microscope for plastic-like features (Norén 2007) or using Raman microspectroscopy (LabRAM HR 800; Horiba Jobin Yvon, Japan). The particle abundance was normalized to the total dry weight of the depth-segment sample.

### Thermoanalytical methods

Fine sediment samples were dry-sieved into four size fractions (2000–500 µm, 500–200 µm, 200 − 50 µm, and 50 − 10 μm) and no additional purification steps were carried out. A practicable subsample of approximately 1 g sediment of each size fraction was weighed in the extraction cells and analyzed in triplicate. First, a polymer extraction on silica gel via pressurized liquid extraction (PLE; 10 mL extraction cells + ASE-350; Dionex, USA) with methanol followed by tetrahydrofuran extraction was performed (Dierkes et al. 2019). Pyrolysis gas chromatography-mass spectrometry (pyr-GC/MS) analysis as described by Dierkes et al. (2019) was performed to quantify the polymer mass of polyethylene (PE), polypropylene (PP), and polystyrene (PS) (LOQ: 0.007 PE mg/g, 0.007 PP mg/g, and 0.008 PS mg/g). In parallel, the silica gel extracts were also used for combustion ion chromatography (C-IC) analysis as described by Kamp et al. (2023) to quantify the polyvinyl chloride (PVC) mass (LOQ: 0.008 PVC mg/g). The workflow enables the quantification of MPs in sediments in a few hours and was referred to as “TAM_sediment_”. The polymer mass was normalized to the corresponding total dry weight of the depth-segment sample.

Additionally, the GF filters first investigated with NIR, being a non-destructive analysis of MPs, were extracted and analyzed with TAM as well (referred to as “TAM_GF-filters_”). If a sample was distributed over multiple GF filters for NIR analysis, all GF filters were combined and extracted together in one batch for TAM analysis. Consequently, the very same sample was complementarily analyzed with both a spectroscopic and a thermoanalytical method.

### Contamination control

To avoid plastic contamination, only cotton lab coats, metal tools, and glassware were used whenever possible, cleaned in an ultrasonic bath and rinsed with deionized water. All devices and work surfaces were carefully cleaned with 30% ethanol solution and deionized water stored in PFA bottles. Chemicals, solutions, and liquids were filtered (0.45 µm cellulose acetate filter) prior to use. Sediment samples were stored in stainless-steel cans and filters in closed glass petri dishes prior to analysis. As procedural blanks (*n* = 7 per analytical workflow), heated (650 °C, 3 h) and calcined sea sand (100–300 µm; Th. Geyer, Germany) was processed and analyzed in the same way as the samples. No significant abundance of MP contamination was detected for the various analytical workflows. This means that for NIR, no MPs were detected in four blanks and only 1–2 MP per sample in three blanks; the total polymer types detected were PP, PS, and PET. For TAM_GF-filters_, the average MP mass in the blanks was 7.4 ± 2.5 mg/kg. The polymer types detected were PP and PVC; in contrast to NIR, PS and PET were not found. For TAM_sediment_, the average MP mass in the blanks was 6.7 ± 3.2 mg/kg. The total polymer types detected were PE, PP, PS, and PVC, but these were not always present in all of the blanks. Full details are provided in SI section S1.5.

## Results and discussion

### MP counts and masses in riverbed sediment profiles

**Fig. 3:**
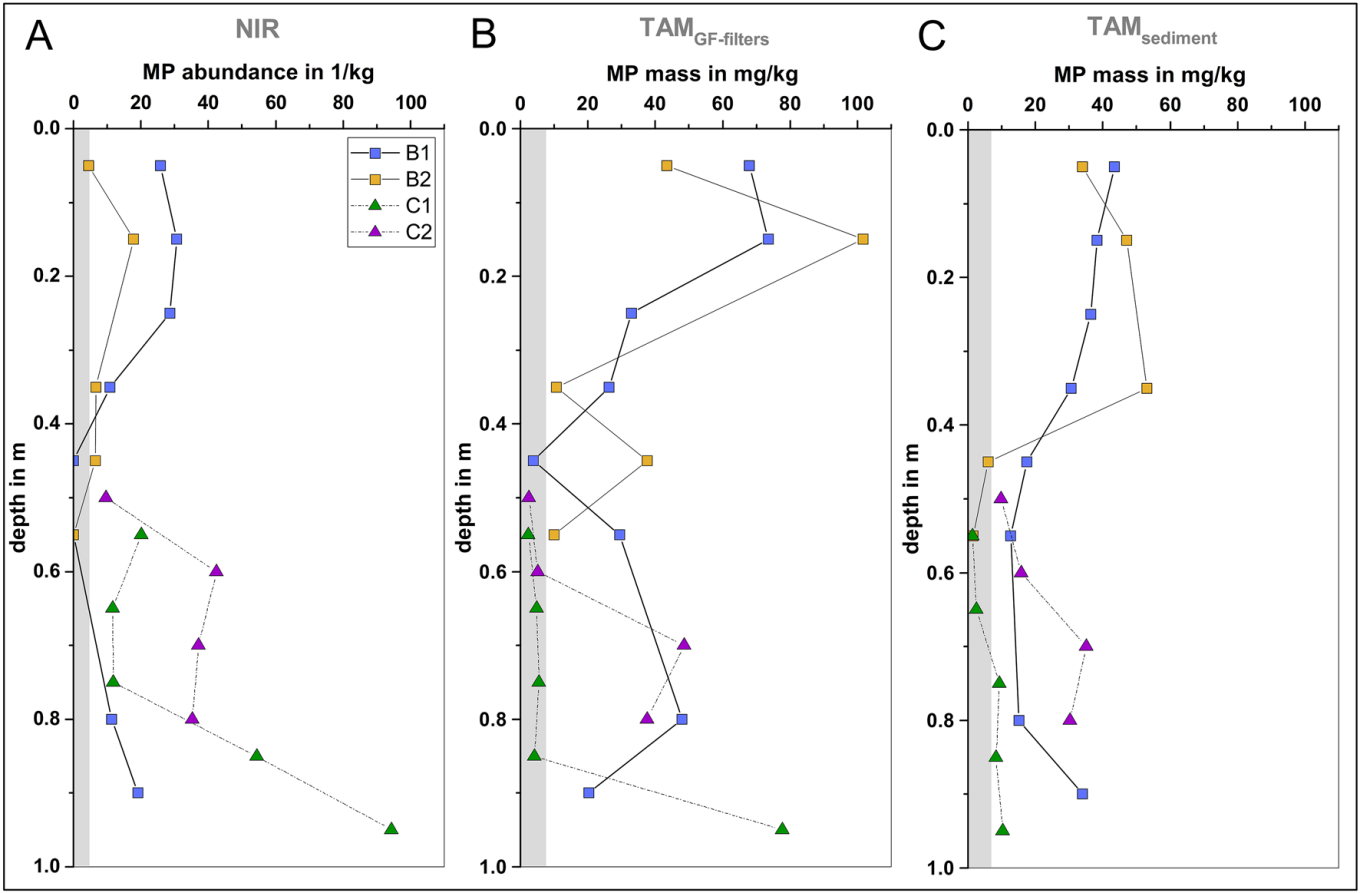
Depth-specific vertical MP concentration profiles for each freeze core resulting from the different analytical workflows; gray-shaded area corresponds to the average procedural blank. Missing data points are due to methodological problems in sieving or sample preparation (see the “Freeze core sampling” section). The sampling depth was ascribed to the midpoint of the sampled 10 cm depth-segment

The vertical MP concentration profiles resulting from the three different analytical workflows are shown in Fig. 3. MPs were detected in all four freeze cores using both analytical methods, NIR and TAM. In one core (C1), MPs were even found down to a depth of 100 cm. This already shows that more shallow investigations would have missed a substantial part of the MPs present in the riverbed sediment, an assessment of which is necessary for the evaluation of microplastic pollution and retention in fluvial systems. The average MP abundance (≥ 100 µm; NIR) was 21.7 ± 21.4 MP/kg (*n* = 22) in total; 12.4 ± 10.5 MP/kg (*n* = 13) in the basin (B1 and B2) and 35.2 ± 25.6 MP/kg (*n* = 9) in the channel (C1 and C2). The absolute number of microplastics identified in each sediment core was 22 MPs in B1, 6 MPs in B2, 57 MPs in C1, and 51 MPs in C2. For identical samples (TAM_GF-filters_), the average MP mass was 31.5 ± 28.0 mg/kg in total, 38.9 ± 27.1 mg/kg in the basin (B1 and B2) and 21.0 ± 25.8 mg/kg in the channel (C1 and C2). The TAM_sediment_ masses are on average 1.5-fold lower compared to TAM_GF-filters_, showing that no MPs were lost during sample preparation for NIR, and that possibly the enrichment of MPs from a larger sample volume via sample preparation (as described in “Near-infrared imaging spectroscopy” section) also improves MPs detection by the TAM.

In this study, the MP concentrations of the shallow riverbed (0–10 cm) ranged from 4.5 to 25.7 MP/kg and 43.4 to ‍67.8 mg/kg, and are in the lower range compared to previous microplastics studies of the Main-Rhine region. Klein et al. (2015) found MPs (63–5000 µm) with mass fractions between 21.8 mg/kg and 932 mg/kg (by weight) and abundances of 228–‍3763 MP/kg (ATR-FTIR) in the first 3 cm of shoreline sediments of the Main and Rhine River. Mani et al. (2019) reported abundances of 260–5970 MP/kg (11–500 µm; µFTIR) in the first 7 cm of midstream sediments of the Rhine River. However, a direct comparison with our study is limited because these studies investigated only shallow sediments, used sampling methods that result in disturbed sample structure, and employed analytical methods with different sensitivities to analyzable particle sizes. Frei et al. (2019) investigated the vertical MP concentration in the headwater sediments of a smallish tributary to the Main River and reported abundances of > 50,000 MP/kg (20–‍500 µm; µFTIR), but for larger MPs (500–5000 µm; ATR-FTIR) only <  < 1 MP/kg. However, no clear overall trend in MP abundances with increasing depth can be identified in these sediment profiles.

Overall, a variable MP concentration profile was identified over sediment depth, with three clear concentration trends. Both analytical methods confirmed these trends, with only minor variations (Fig. 3 and SI Fig. S3): (1) Fairly constant concentrations were found averaging ~ 22.6 MP/kg or ~ 58.7 mg/kg in the top layers (0–‍30 cm); (2) a substantial decrease to ~ 6.5 MP/kg or ~ 16.7 mg/kg was found in the middle layers (30–‍60 cm); (3) a strong increase was detected from lower concentrations in the middle layers to ~ 36.0 MP/kg or ~ 34.9 mg/kg in the deep layers (60–‍100 cm), which is in the same order of magnitude or even higher than in the top layers. Although TAM_sediment_ masses were lower compared to TAM_GF-filters_, the overall trend is well represented, too. These similar vertical trends in MP mass and abundance indicate a good overall inter-comparability of the obtained results. Other studies investigated vertical MP profiles in riverbeds up to a depth of 50–60 cm only, and reported both decreasing as well as increasing trends in MP abundance with sediment depth (overview in SI Table S5). Variations in sampling site characteristics (e.g., hydrological conditions, sediment texture, or riverbed morphology) and the diversity of samples could be several possible reasons for the different observed trends. However, information on hydrogeological and hydrological boundary conditions or sediment texture are often not provided and hamper the direct comparison of studies.

**Fig. 4:**
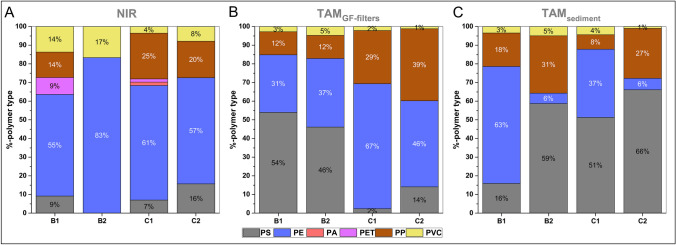
Relative polymer composition for each freeze core resulting from the different analytical workflows. Please note that results from NIR represent MP numbers in a sample, while TAM represent MP mass of a sample

The relative polymer composition for each freeze core and analytical workflow is shown in Fig. 4. In total, six different synthetic polymer types (PS, PE, PP, PVC, polyethylene terephthalate (PET), and polyamide (PA)) were detected using NIR. TAM detected all four polymer types (PS, PE, PP, and PVC) that could be identified using this method (see the “Thermoanalytical methods” section). Thus, the smaller amounts of PA (2%) and PET (2–9%) in B1 and C1 could be identified only by NIR. The dominant polymer types PE, PP, and PS made up > 75% of microplastics identified in the riverbed sediments via both analytical methods. This is in good accordance with findings that PE, PP, and PS are predominant in river water and sediments of the Rhine-Main region (Schrank et al. 2022; Mani et al. 2019; Klein et al. 2015), and similar to other German regions (Munz et al. 2023; Schrank et al. 2022; Laermanns et al. 2021). Both analytical methods detected overall the same dominant polymer types, with partial variations between cores. NIR indicated PE as the most common polymer type in all cores, but TAM_GF-filters_ detected higher proportions of PS for B1 and B2, and TAM_sediment_ detected higher proportions of PS in B2, C1, and C2 (Fig. 4). For TAM_sediment_, the differences in relative polymer composition are likely due to the small and maybe non-representative sample volume of 1 g fine sediment compared to 150 g fine sediment processed for TAM_GF-filters_. However, despite the small sample volume and lack of sample purification steps performed, the dominant polymer types and general vertical trends of MP concentration were consistently identified by TAM_sediment_. Consequently, thermoanalytical methods seem suitable as a fast-screening method before a detailed analysis of selected samples with more complex and time-consuming spectroscopic methods.

Although the MP concentrations found in this study are in the lower concentration range compared to other studies, the detection of MPs in the entire sediment core up to 100 cm depth emphasizes the substantial and widespread MP pollution of riverbed sediments. This supports the assumption that river sediments can represent an accumulation zone and temporal sink for MPs in fluvial systems (Drummond et al. 2022; Margenat et al. 2021).

### MP concentration vs. sediment properties

**Fig. 5:**
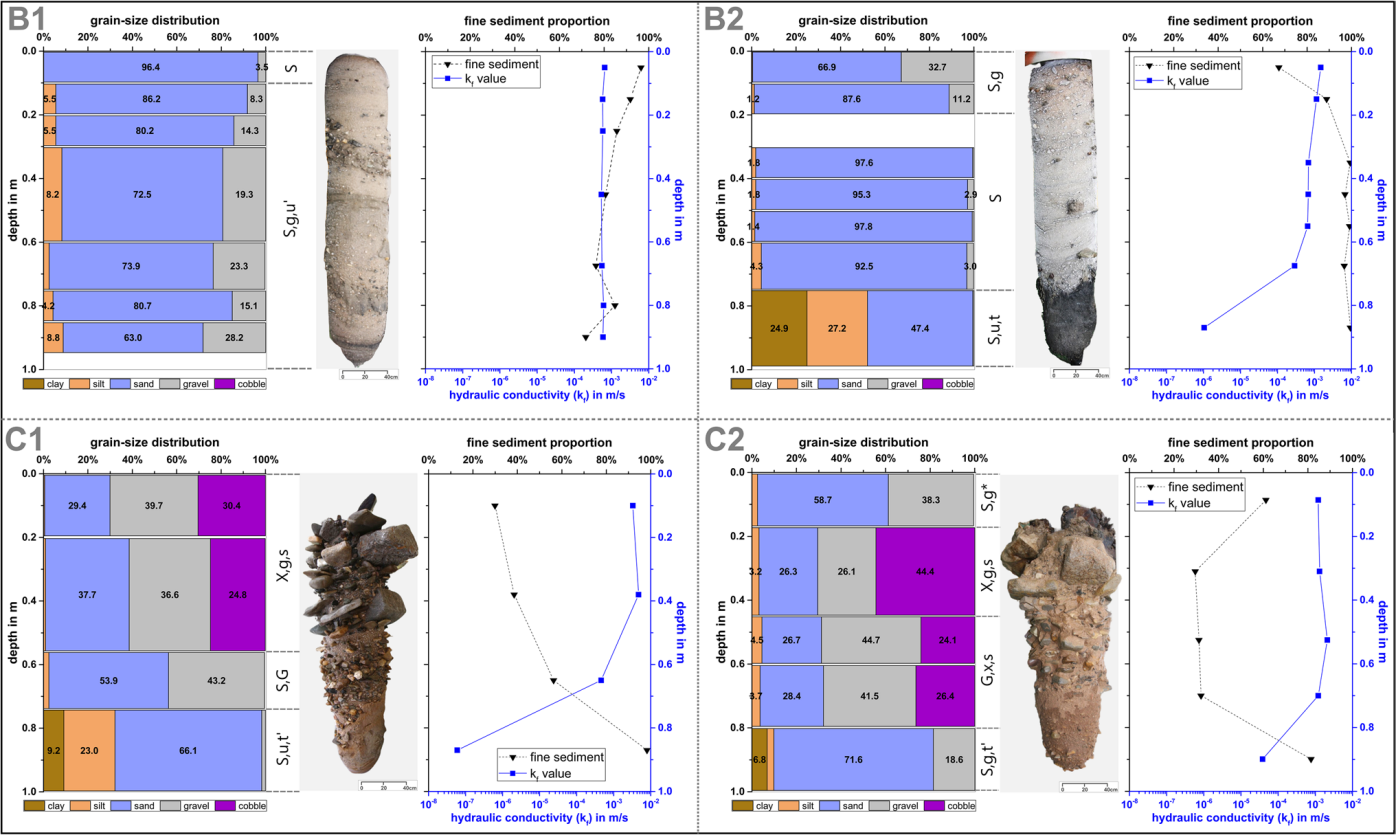
Summary of sediment properties for all freeze cores (B1, B2, C1, and C2). Sediment grain-size distribution corresponds to cobble “x” (> 63 mm), gravel “g” (> 2.0–63 mm), sand “s” (> 0.063–2.0 mm), silt “u” (> 0.002–‍0.063 mm), and clay “t” (≤ 0.002 mm) (ISO 14688–1 2017). On the right are the profiles of the proportion of fine sediment (< 2 mm) and the hydraulic conductivity (*k*_f_)

The analyzed sediment properties of the extracted riverbed freeze cores are summarized in Fig. 5. The riverbed material of the river basin (cores B1 and B2) is characterized by a sand-gravelly sediment texture and a high proportion of fine sediment (< 2 mm) at 88.1% on average, ranging from 67.3 to 99.5%. First, a relatively homogeneous layer (0–‍80 cm) with hydraulic conductivity (*k*_f_) values ranging from 5.32 × 10^−4^ to 1.48 × 10^−3^ m/s and an organic matter content of 4–13 g/kg was identified. At the bottom (80–100 cm), in parts an organic rich layer (organic up to 107 g/kg) with *k*_f_ values ranging from 1.05 × 10^−6^ to 5.92 × 10^−4^ m/s was found. The riverbed material of the shipping channel (cores C1 and C2) is dominated by sandy gravel sediments and a lower proportion of fine sediment at 44.6% on average, ranging from 29.0 to 81.4%. First, armourstones dominated down to a depth of 40–‍50 cm (*k*_f_ values of 1.20 × 10^−3^–4.70 × 10^−3^ m/s), followed by gravelly sediments (*k*_f_ values of 4.60 × 10^−4‍^–‍4.68 × 10^−3^ m/s) at a depth of 50–‍80 cm, and at the bottom (80–‍100 cm) sandy sediments (*k*_f_ values of 6.00 × 10^−8^–‍3.80 × 10^−5^ m/s). The organic matter content of cores C1 and C2 ranged from 3 to 12 g/kg.

It is assumed that the transport of MPs in fluvial systems follows a similar trajectory as that of natural particles (Hoellein et al. 2019), and that principles of fine sediment (< 2 mm) dynamics can be transferred to MPs (Waldschläger and Schüttrumpf 2020). So, the proportion of fine sediment can possibly be used as an indicator of riverbed flow dynamics and as a key factor in retention and relocation of MPs in riverbed sediments (Margenat et al. 2021; Waldschläger and Schüttrumpf 2020). Until now, only few freshwater studies have been published that investigated both depth-specific MP concentration profiles and sediment properties (e.g., sediment grain-size analysis) (SI Table S5). Corcoran et al. (2020) found more MPs in fine-grain sizes in top-layer sediments of the Thames River, but they did not look at the relationship across different sediment depths. Instead, we found on average more MP per kg in sediment cores from the channel (C1 and C2) than from the basin (B1 and B2). Overall, there was no significant linear relationship between the depth-specific MP abundance or masses and the corresponding proportion of fine sediment (*R*^2^ = 0.09 or *R*^2^ = 0.14), estimated *k*_f_ values (*R*^2^ = 0.06 or *R*^2^ = 0.007), or organic matter content (*R*^2^ = 0.01 or *R*^2^ = 0.04) of the riverbed (SI Fig. S5). These findings are in accordance with Mani et al. (2019), who also found no significant linear relationship (*R*^2^ = 0.22) between MP concentrations and fine sediment proportions in riverbed sediments of the Rhine River. Consequently, the fine sediment proportion could not be used as a direct proxy of the MP distribution in riverbed sediments. In addition, it is necessary to delineate which principles from fluvial sediment transport or the sedimentation dynamics of natural sediments are really transferable to microplastics (Waldschläger et al. 2022). For example, sediment core B1 shows a nearly uniform distribution of fine sediment proportion over depth (Fig. 5), but a characteristic depth-specific MP concentration (SI Fig. S3A). We hypothesize that the depth-specific MP distribution can provide complementary information to findings derived from fine sediment proportion, and enables more detailed conclusions about the sediment dynamics and accumulation processes within the last decades (see the “Microplastics as a process marker in riverbed sediments” section next).

### Microplastics as a process marker in riverbed sediments

**Fig. 6:**
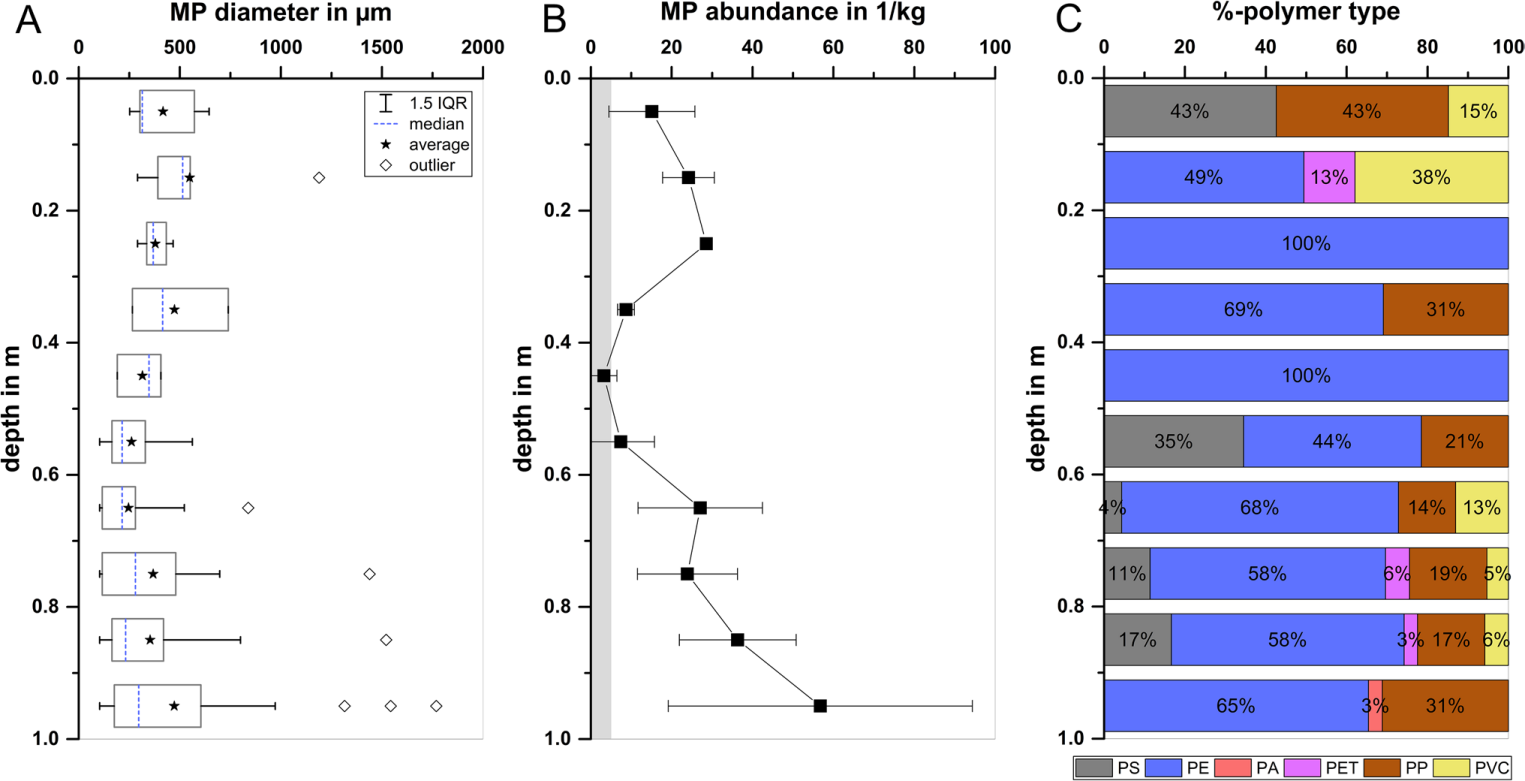
Combining all sediment cores: **A** depth-specific MP size distribution, shown as a boxplot of all MPs found in the same depth-segment; **B** depth-specific mean MP abundance per depth-segment, gray-shaded area corresponds to the mean procedural blank; **C** depth-specific mean polymer type fraction per depth-segment

The depth-specific polymer type distribution can be an important indication on the one hand for stratigraphical deposition and distinction between younger and older sediments, and on the other hand for particle relocation processes over sediment depth. Due to the exponential growth in global plastic production since the 1950s (PlasticsEurope 2022), MPs might be used as a general marker for sediment depositions after 1950 and as a specific marker (1910–1990) based on the age of earliest possible occurrence (EPO age) for each polymer type (Weber and Lechthaler 2021). We found different polymer types at all depths up to 100 cm, but no clear pattern or trends in the polymer composition for establishing a stratigraphy (Fig. 6C and SI Fig. S4). C2 shows a similar polymer composition at all depths and B2 is dominated by PE. In B1 and C1, polymer types (PP and PET) with younger EPO ages (1954 and 1973) were found in deeper sediment layers than were polymer types (PVC and PS) with older EPO ages (1912 and 1931). Furthermore, we identified low-density, buoyant polymer types (PP and PE) mainly in deep layers (> 50 cm). This finding is contrary to the intuitive expectation that only MPs with a high specific density have the potential to sink out of the water column and infiltrate the sediment driven by hydrodynamic forces. However, the abundance of buoyant polymer types in river sediments has already been revealed in previous studies (Bao et al. 2023; Klein et al. 2015; Frei et al. 2019). This could be due to the fact that biofouling, impurities, weathering, sorption processes, and hetero-aggregation can increase particle density and promote the sinking and sedimentation of MPs. Based on the polymers with younger EPO ages (PET, 1973) detected in deep layers (> 50 cm), an undisturbed natural sedimentation can be excluded and it is a strong indication that in situ relocation processes overlayed the sediment stratigraphy at the study site after 1970.

Another important clue for particle retention processes can be the depth-specific MP concentration profiles. In river sections with permanent losing conditions, as prevailing in our study area, a significant input of MPs into the riverbed through advection and a decrease in MP concentration with increasing sediment depth through particle retention processes are assumed. At our study site, average MP concentrations decreased from ~ 22.6 MP/kg in the top layers (0–‍30 cm) to ~ 6.5 MP/kg in the middle layers (30–60 cm) (Fig. 6B), highlighting the substantial MP retention in top-layer sediments. Laboratory column tests of MP retention in sand and gravel sediments confirmed the decrease in MP concentration with increasing depth due to particle filtration. However, in the gravel-packed columns, MPs < 500 µm were relocated to a sediment depth of 50 cm, while MPs > 500 µm were retained in the first 30 cm of the column sediment (Munz et al. 2024).

In the sediment cores, the MP sizes found ranged from 104 to 1770 µm in diameter and show a characteristic depth-specific distribution (Fig. 6A). In the top sediment layers of 0–30 cm, only MPs between 200 µm and 1200 µm in diameter were found. The relative proportion of the MP fraction < 500 µm increased up to a depth of 50–60 cm, and this fraction accounted for the largest proportion of particle sizes in deep layers (60–‍100 cm). In depth-segments B1 (40–60 cm) and B2 (50–60 cm), no MP abundance, but a significant MP mass ranging from 3.7 to 29.5 mg/kg was found (Fig. 3). While NIR has been limited to the analysis of particles with diameters ≥ 100 µm, TAM detects the cumulative mass of all particles in a sample and is limited only by the sample processing steps (here 6 µm filtration), besides the limit of quantification (LOQ). Consequently, the proportion of small-sized MPs < 100 µm seems to increase downward, partly reaching 100% in individual mid-layers of B1 (40–‍60 cm) and B2 (50–60 cm) and accounting for a considerable proportion of the MP mass. According to laboratory column experiments, a ratio of MP size (*d*_MP_) to sediment grain-size (*d*_sed_) smaller than 0.11 is required for MPs to infiltrate through the sediment (Waldschläger and Schüttrumpf 2020; Munz et al. 2024). Applied to our sediment properties (median sediment grain size: *d*_50_ = 0.65 mm), only MPs < 67 ± 23 µm in diameter are able to infiltrate down to the deep-layer sediments. These findings are consistent with other riverbed studies, which also found an overall decrease in vertical MP concentration in shallow layers (0–‍60 cm), but an increase in the percentage of small-sized MPs over sediment depth (Niu et al. 2021; Zhou et al. 2021; Fan et al. 2019). These particle distribution patterns are characteristic for particle transport and retention processes in riverbed sediments. The small-sized MPs tend to be mobile, while larger particles will be preferentially retained in shallow layers (0–‍30 cm). Nevertheless, in the combined profile, the highest MP abundances and largest MPs in terms of size (Fig. 6) were found in the deep layers (60–‍100 cm) of the riverbed. For our sediment properties, MPs with a diameter > 194 ± 92 µm are very unlikely to infiltrate substantially into the riverbed (*d*_MP_/*d*_sed_ > 0.32) and are retained in shallow layers (Waldschläger and Schüttrumpf 2020; Munz et al. 2024). Furthermore, these large MPs clearly exceed the maximum range of 100–‍300 µm in pore size distribution of coarse sand sediments (Minagawa et al. 2008; Horoshenkov and Mohamed 2006). Transport through the sediment pore space via infiltrating surface water can therefore be ruled out as a mechanism. Disturbance of the sediment by flooding, mechanical influences (e.g., shipping traffic), benthic bioturbation, or construction work are possible mechanisms for the relocation of MPs into deeper sediment layers. Due to regulation by weirs, there are no rapid changes in water level at the study site. In the last 20 years before sampling, no extreme flooding and only two high water level events with approximately 100 cm water level change were recorded. Therefore, water level changes can probably be ruled out as a potential explanation for MP relocation to deeper sediments. Discharge at the study site can reach up to ~ 800 m^3^/s (MQ = 117 m^3^/s) and flow velocity up to 1.3 m/s (MQ = 0.45 m/s) caused by annual flood events. Due to bank and bottom protection for inland waterways, it is unlikely that yearly flood events or ship-induced waves and flows affect sediment depths of more than a few decimeters (BAW 2010). However, such dynamics can lead to a resuspension and remobilization of already-deposited fine sediment in the uppermost decimeters of the riverbed (Waldschläger et al. 2022). Disturbance-induced resuspension can facilitate the remobilization of microplastics from the sediment into the overlying water, leading to a reduction in the abundance of MPs in the top layers of the riverbed sediment (Xia et al. 2021). This can be one possible explanation for the lower MP concentrations in the top 20 cm of the riverbed (Fig. 6). In pure infiltration-affected sediments, an exponential decrease in MP abundance with depth would be expected (Munz et al. 2024), with the highest abundances in the uppermost layers. Finally, the fairly constant MP concentrations in combination with the large variety of polymer types and sizes within the uppermost layer (0–30 cm) indicate a substantial hydrodynamic disturbance or displacement (i.e., sediment mixing and bedload transport processes caused by water flow). Such dynamics could also hamper the permanent deposition of large amounts of MPs into the sediment and be one possible reason for the overall low microplastics contamination of the riverbed sediment compared to other studies (“MP counts and masses in riverbed sediment profiles” section and SI Table S5). Benthic bioturbation processes would result in a more gradual MP distribution (Näkki et al. 2017; Xue et al. 2020) and could not explain the low MP concentration in the middle layers (30–60 cm). Therefore, artificial relocation, e.g., by construction works performed in previous decades (“Study site” section), seems to be the most plausible explanation for the detection of large MPs (> 1 mm) and younger polymer types (e.g., PET) in deep-layer sediments (60–100 cm). A detailed chronological documentation of past hydraulic construction works is not available, but significant river morphology-altering works can be excluded in recent decades. Once deposited in the deep-layer riverbed sediments, the MPs are immobilized and remain there over decades due to their high persistence.

### Conceptual approach of riverbed dynamics and its limitations

**Fig. 7:**
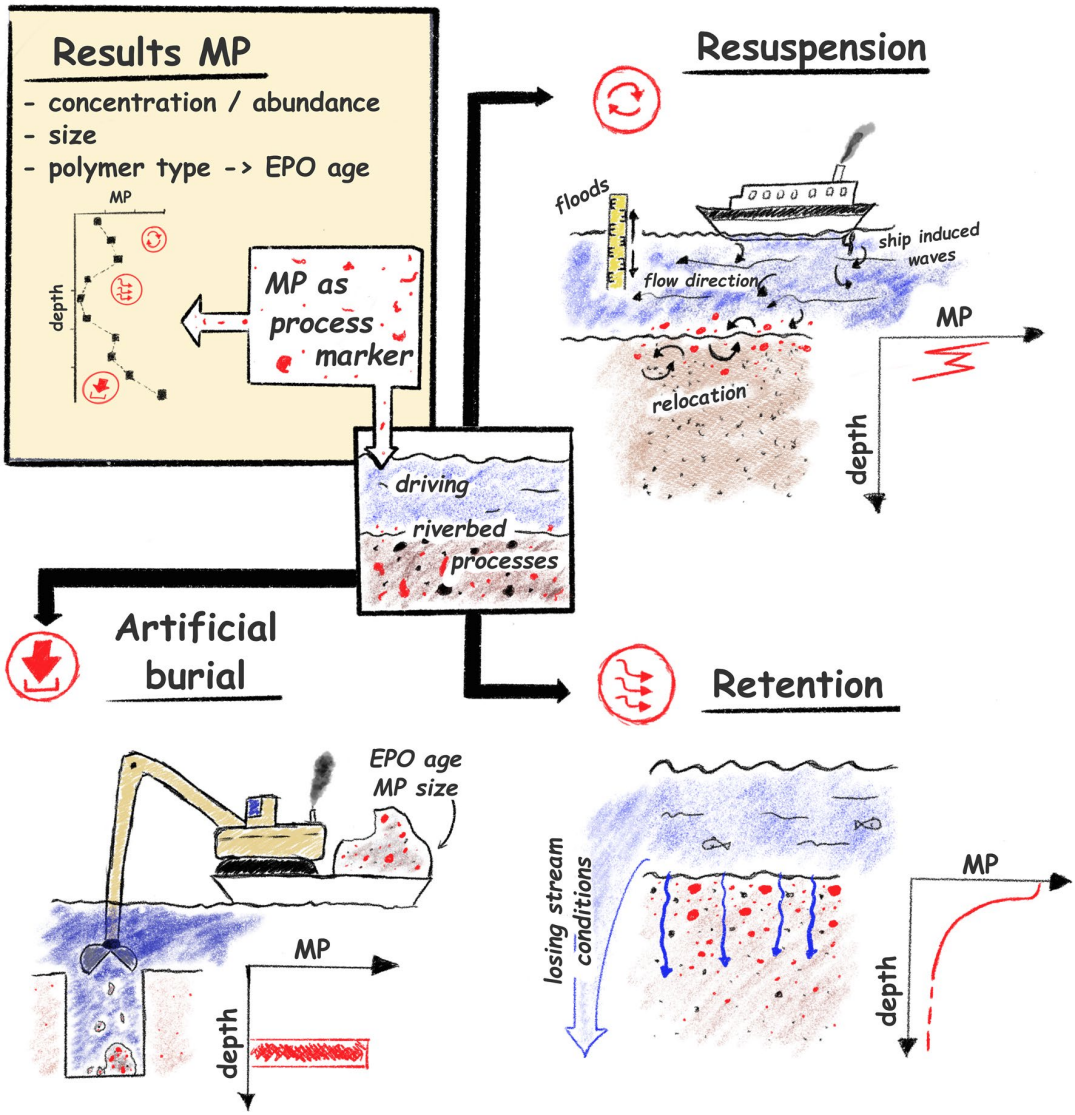
Graphical illustration of an initial conceptual approach to using the depth-specific microplastics distribution as a potential process marker to derive the driving riverbed processes

Considering all our findings, we propose that the distinctive depth-specific profile of identified MPs (Fig. 6) indicates a complex interplay of factors controlling the dynamics of riverbed sediments. We hypothesize that these factors include a combination of (1) resuspension and relocation processes of fine material in the top layers (0–‍30 cm) due to mechanical disturbance (e.g., ship-induced waves, flood events, or bioturbation); (2) particle retention processes in shallow and middle layers (0–60 cm); and (3) artificial burial from previous construction work in deep layers (60–100 cm). We summarize this conceptual approach in a graphical representation in Fig. 7.

Nevertheless, it should be noted that non-uniform sedimentation processes and water dynamics may also contribute to the complex depth distribution of MPs. Therefore, this initial approach should not be interpreted as universal, since several assumptions had to be made and there are some limitations and restrictions in the conceptual realization. In this study, a sampling site within a better-known and more controlled hydrological situation was considered (“Study site” section), which is essential to derive more straightforward specific processes from the MP distributions found. However, information on boundary conditions, such as the MP concentration in the river water as a temporally variable input source or detailed knowledge of sediment formation by chronological dating analysis, is still missing and assumptions had to be made. Furthermore, processes such as the degradation of microplastics in the sediment by mechanical weathering and microbial colonization, which may distort the MP size distribution, could not be examined.

It is also important to be aware of the methodological challenges and difficulties in handling and preparing the sediment samples (see the “Freeze core sampling” section). By excluding the highly organic and cobble-dominated sediment layers, potentially crucial information that can influence the comparison of sediment properties with the depth distribution of MPs may be lost. In the top layers of cores C1 and C2, insufficient fine sediment could be extracted for analysis due to the high-volume fraction of cobbles, but no MPs were found through visual inspection of the cobbles. Therefore, it can be roughly assumed that the MP concentration in these top layers is low if related to the total sediment mass. In the deep layers of core B2, with a high proportion of organic material, the MP abundance could indeed not be determined. Nevertheless, the four sediment cores exhibited comparable trends in MP distribution, suggesting that the results are sufficiently reliable to draw initial conclusions about the processes occurring within the riverbed.

Another limitation concerns the analytical methods used. NIR was limited to the analysis of particles ≥ 100 µm in size, missing a substantial proportion of the small MP fraction (< 100 µm), and TAM offered limited information on particle size and type. The number of small MPs (< 100 µm) may be important to assess the total MP contamination of the sediment or in terms of toxicity and health relevance. However, in order to draw conclusions on the sediment dynamics of the riverbed, the depth-specific distribution of larger MPs (≥ 100 µm) provides valuable information on riverbed dynamics such as relocation and redeposition processes, as already discussed in the “Microplastics as a process marker in riverbed sediments” section. Information on the depth-specific composition of as many different polymer types as possible is important for determining chronological deposition processes and dynamics. Consequently, at least complementary information on the depth-specific number, size, and polymer type of MPs is necessary to draw conclusions on particle retention or relocation processes. In contrast to spectroscopic techniques such as NIR, TAM can only provide limited information on MP masses for specific size classes, and only if size-fractionated sample preparation steps (e.g., sieving) have been performed. However, TAM_sediment_ is suitable as a fast-screening method prior to a detailed analysis using more complex and time-consuming spectroscopic methods. The depth-specific distribution of MP mass and limited information on particle size and polymer type already provide an indication of the maximum sediment depth of anthropogenic influence and the microplastics load of the sediment. Ideally, the two techniques should not compete with each other, but are performed sequentially or in parallel aliquots to provide the most comprehensive information. Despite these limitations, the study provides valuable initial information on the occurrence and distribution of microplastics in deep layers of the riverbed.

### Recommendations for interdisciplinary implications and further research

Furthermore, the interdisciplinary application of MPs as a novel artificial marker for questions in the sedimentary context could be demonstrated. In particular, knowledge of the geohydraulic properties and sedimentary structure of the riverbed is required to assess the effects of hydraulic engineering on the interaction between surface water and groundwater. Many different established hydrogeological methods (e.g., grain-size analysis, in situ permeameter/hydraulic tests or tracer tests) are available to characterize the riverbed sediment, but all have restrictions and limited applicability (e.g., disturbed sediment structure or scale dependency). Combining established exploration methods (e.g., freeze core technique and grain-size analysis) with complementary analysis of MP distribution can be a comparatively inexpensive and rapid approach with which to improve and enhance the knowledge of geohydraulic sediment properties. Based only on the depth-specific distribution of MP abundance, size, and type, without additional information on hydrological conditions or sediment structure, it is already possible to estimate the maximum sediment depth of anthropogenic influence, identify particle accumulation and retention zones, and determine chronological sediment deposition in the riverbed.

However, fluvial systems are complex and can be highly dynamic, resulting in a complex interplay and superposition of multiple processes that can influence the dynamics of riverbed sediments. Currently, there is a lack of knowledge regarding the distribution, retention, and relocation potential of MPs in riverbeds of different systems and under uncontrolled hydrological situations. Consequently, further investigation is required to determine the influence of disturbance-related processes, such as turbulence resulting from fluctuating energy levels (e.g., storms or strong currents), bioturbation or resuspension, on the vertical MP distribution. This is particularly important when several of these driving factors are superimposed, as it is challenging to distinguish between distinct MP distribution patterns of individual sediment processes. Therefore, more attention is required to identify depth-specific MP patterns, which are characteristic of distinct processes in the riverbed. Extensive laboratory column experiments can further improve our understanding of the vertical transport and retention processes of MPs of various sizes and types in different saturated porous media. Additionally, the implementation of field studies at a greater number of sites, encompassing a wider range of sediment types and hydrological boundary conditions, would provide a more comprehensive understanding of the depth-specific MP distribution within riverbeds. Taking all this information together can further improve our understanding of the driving processes and offer a conceptual approach to using microplastics as a process marker for sediment dynamics.

## Conclusion

This study provides insight into the depth-specific occurrence and composition of MPs (≥ 100 µm) in deep-layer riverbed sediments down to a depth of 100 cm. MPs were detected over the entire riverbed depth, with PE, PP, and PS as the dominant polymer types. Three distinct vertical concentration trends could be identified, a fairly constant concentration in the top layers (0–30 cm), a decrease in the middle layers (30–60 cm), and a strong increase from the middle to the deep layers (60–100 cm). Based on this depth-specific MP distribution, the potential of microplastics to be used as a process marker was elucidated to infer the processes controlling sediment dynamics in the riverbed. The conceptual approach presented here highlights the use of microplastics, an anthropogenic pollutant that is ubiquitous and persistent in riverine environments, as a versatile artificial tracer. The approach has the potential to be applied to other river sections and sediment structures, provided that microplastics are present in river water.

### Supplementary Information

Below is the link to the electronic supplementary material.

Supplementary file1 (PDF 1.92 MB) Additional details on the sampling method and sampling site characteristics including the hydrogeological conditions are provided here. This includes a tabular summary of studies investigating vertical microplastics concentration profiles in fluvial sediments. Detailed results of procedural blanks and additional illustrations of depth-specific microplastics concentration profiles are also given.

## Data Availability

All datasets generated or analyzed during the current study are available from the corresponding author on reasonable request.
