# Supplementary material for: Riverbed depth-specific microplastics distribution and potential use as process marker
Source: Environ Sci Pollut Res Int. 2024 Jul 4;31(32):45326–40. doi: 10.1007/s11356-024-34094-z (PMC11255049; doi:10.1007/s11356-024-34094-z)
Supplement: Supplementary file 1 — Supplementary file1 (PDF 1.92 MB) Additional details on the sampling method and sampling site characteristics including the hydrogeological conditions are provided here. This includes a tabular summary of studies investigating vertical microplastics concentration profiles in fluvial sediments. Detailed results of procedural blanks and additional illustrations of depth-specific microplastics concentration profiles are also given. [file 11356_2024_34094_MOESM1_ESM.pdf]

# – Supporting Information –

Riverbed depth-specific microplastics distribution and potential use as process marker

Marco Pittroff [1,2], Constantin Loui[2], Sascha E. Oswald[2], Mathias Bochow[4], Jan Kamp[3], Georg Dierkes[3], Hermann-Josef Lensing[1], Matthias Munz[2]*

[1] Department Geotechnical Engineering, Federal Waterways Engineering and Research Institute (BAW), Kußmaulstraße 17, 76187 Karlsruhe, Germany

[2] Institute of Environmental Science and Geography, University of Potsdam, Karl-Liebknecht-Str. 24-25, 14476 Potsdam, Germany

[3] German Federal Institute of Hydrology, Am Mainzer Tor 1, 56068 Koblenz, Germany

[4] Helmholtz Centre Potsdam, GFZ German Research Centre for Geosciences, Section 1.4 Remote Sensing, Telegrafenberg, 14473 Potsdam, Germany

* Corresponding author email: pittroff.marco@gmail.com

S1 Supplementary Materials and Methods

S1.1 Sampling site - Hydrogeological conditions

The Quaternary (~25–30 m thickness) is built of fluviatile gravels and sands and formed a porous aquifer with a hydraulic conductivity (k_f) of $1.5x10^{-3}$–$5x10^{-4}$ m/s (Fig. S1). The underlying Middle Muschelkalk (~40 m thickness) is built of gypsum and rock salt deposits fully depleted and formed a highly permeable karst aquifer with high variable k_f values of $1.5x10^{-3}$–$6x10^{-5}$ m/s. The Upper Muschelkalk on the eastern and western edge (~20–25 m thickness) formed a fissure aquifer with k_f values of ~$1.0x10^{-6}$ m/s.

In the Main valley, several wells for water supply are operated in the area of the Marktbreit barrage near the towns of Sulzfeld and Marktsteft. Two horizontal filter wells and one vertical filter well operates (~260,000 m³/month) about 100–160 m from the Main River developed in the quaternary aquifer. The horizontal filters partly reach up to 75 m to the river. The general hydrological conditions can be described as follows (Fig. S1). Water from the Quaternary aquifer flows towards the wells on both sides of the river, while river water infiltrated into the aquifer (bank filtration). Additionally, water from the Muschelkalk discharges into the quaternary aquifer from the outwards side.

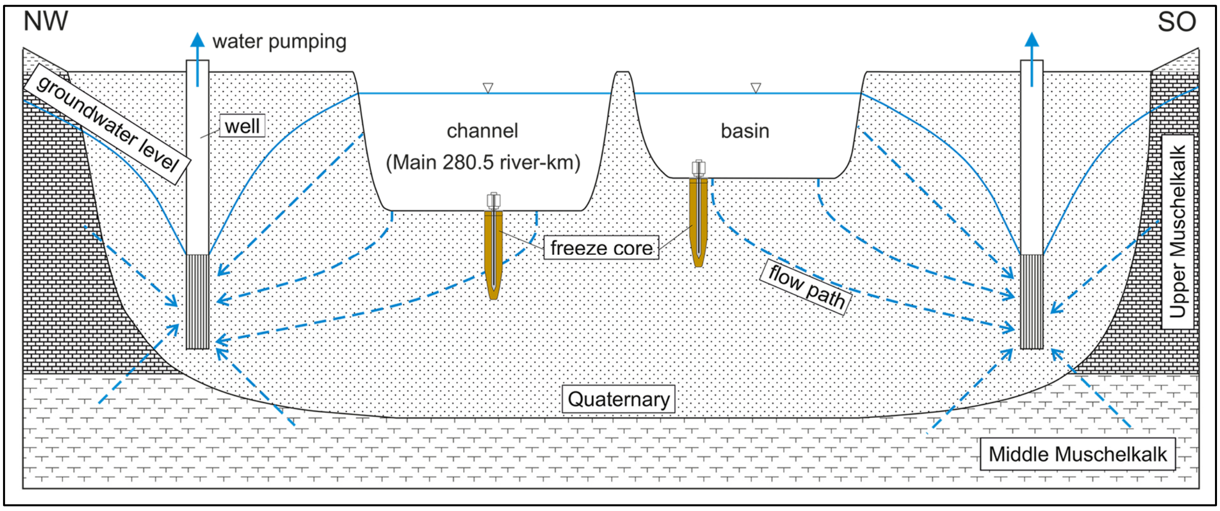

Fig. S1 *Schematic hydrogeological cross-section (not size-scaled) from northwest to southeast through the sampling site (280.5 river-km) representing the estimated hydrological situation of a losing stream with infiltrating river water (blue dashed arrows). The basin is connected to the channel, i.e., the shipping waterway, though not shown in this cross-section*

S1.2 Content of organic matter

The content of organic matter in sediment samples was determined using the loss on ignition (LOI) according to DIN 18128. In brief, the test consists of placing 30 to 1000 g of dried and crushed sediment in a tared crucible and heating in a temperature-controlled furnace at 550 °C for 4–8 h. The amount of organic matter is calculated by comparing the weight of the sample before and after heating. The organic matter content was normalized to the dry weight of the depth-segment sample.

Table S1 *Overview of the estimated organic matter content in sediment freeze cores per depth-segment*

Sediment core	Depth in m	Organic matter content in %-mass	Organic matter content in g/kg
**B1**	0.00–0.10	0.5	5
	0.10–0.30	0.6	6
	0.30–0.75	1.1	11
	0.75–0.85	0.4	4
	0.85–0.95	0.5	5
**B2**	0.00–0.30	0.6	6
	0.30–0.75	1.3	13
	0.75–1.00	10.7	107
**C1**	0.00–0.55	-*	-*
	0.55–0.75	0.3	3
	0.75–1.00	1.2	12
**C2**	0.00–0.50	-*	-*
	0.50–0.80	0.3	3
	0.80–1.00	1.1	11

*Not analyzed

S1.3 Sampling method: Freeze core technique

The used freeze-core sampling technique is based on the principle of Stocker and Williams (1972) and allows sampling of cohesionless sediments by freezing while preserving the sediment structure. A tapered, internally hollow freezing lance with a diameter of 4.5 cm and a length of 100 cm was used and extended by several metal pipes with an internal nitrogen line. The lance was lowered from a ship vertically and carefully, to prevent suspension of fine particles, to the bottom of the river bed. The freezing core lance is gradually inserted into the sediment using a falling weight. Subsequently, the nitrogen pressure vessel is connected to the internal nitrogen line and liquid nitrogen (-196°C) flows through (30–40 min), vaporizes, and escapes back into the atmosphere via the extension tube (Fig. S2). Thereby the surrounding sediment freezes to the lance and forming a solid core. Depending on the sediment grain-size distribution, freezing duration, nitrogen flow rate, and natural temperature of ground- and surface-water, the diameter of the frozen cores ranging from 20 cm to 40 cm. The consumption of liquid nitrogen was approx. 40 L per core. More details on the methodology are described in Strasser et al. (2015) and Straßer et al. (2014).

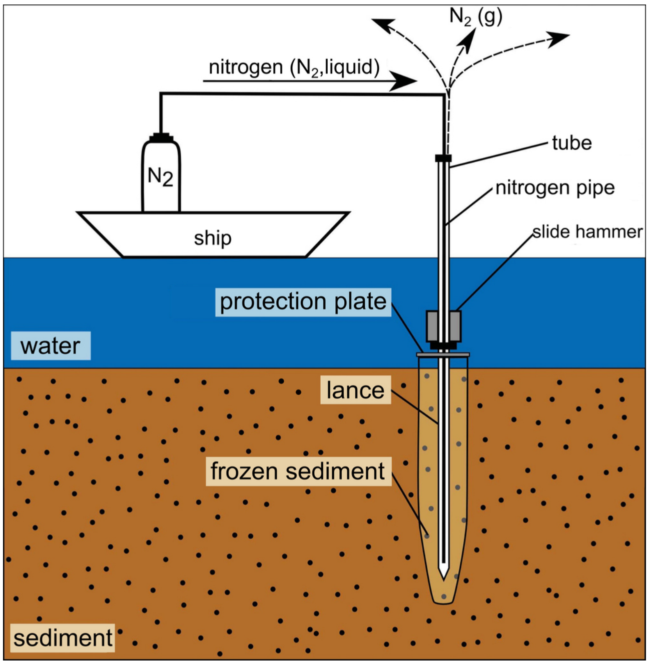

Fig. S2 *Principle of freeze core sampling of riverbed sediments from a boat at high water levels and deep sediment (modified from Straßer et al. (2014))*

S1.4 Near infrared (NIR) imaging spectroscopy

For MP identification the PlaMAPP (Plastic Mapper) algorithm was used, which is mainly based on a penalty score calculated as the spectral distances between absorption bands of the recorded pixel and reference spectra (Schmidt et al. 2018, Munz et al. 2023). Polymer identification was done by automatically matching recorded pixel spectra with a reference library containing the most common polymer types, as well as organic materials. The method allows to identify the following polymer types: polyethylene (PE), polypropylene (PP), styrene polymers (PS) (as a group consisting of polystyrene, acrylonitrile-butadiene-styrene and styrene-acrylonitrile), polyvinyl chloride (PVC), polyethylene terephthalate (PET), ethylene-vinyl acetate (EVAC) and ethylene vinyl alcohol (EVAL) (as a group), polyamide (PA), polycarbonate (PC), polyurethane (PU), polyoxymethylene (POM), and polymethyl methacrylate (PMMA).

S.1.5 Contamination control

Table S2 *Overview of procedural blanks of the NIR workflow*

Sample	MP per sample	MP per kg	Polymer type	Diameter in µm
**blank 1**	0	0	-	-
**blank 2**	0	0	-	-
**blank 3**	0	0	-	-
**blank 4**	0	0	-	-
**blank 5**	1	6.7	PP	520
**blank 6**	2	13.3	PS	187
			PP	164
**blank 7**	2	13.3	PET	303
			PP	214
**Mean ± SD**	**0.7 ± 0.9**	**4.8 ± 5.9**		

Table S3 *Overview of procedural blanks of the* $TAM_{GF\text{-}filters}$ *workflow*

Sample	PE conc. in mg/kg	PP conc. in mg/kg	PS conc. in mg/kg	PVC conc. in mg/kg	Total conc. in mg/kg
**blank 1**	0.0	4.8	0.0	0.7	5.5
**blank 2**	0.0	9.7	0.0	0.6	10.4
**blank 3**	0.0	3.2	0.0	0.5	3.6
**blank 4**	0.0	4.8	0.0	0.6	5.4
**blank 5**	0.0	7.7	0.0	0.7	8.4
**blank 6**	0.0	7.2	0.0	0.8	7.9
**blank 7**	0.0	10.1	0.0	0.8	10.8
**Mean ± SD**	**0.0**	**6.8 ± 2.4**	**0.0**	**0.7 ± 0.1**	**7.4 ± 2.5**

Table S4 *Overview of procedural blanks of the TAM$_{sediment}$ workflow*

Sample	PE conc. in mg/kg	PP conc. in mg/kg	PS conc. in mg/kg	PVC conc. in mg/kg	Total conc. in mg/kg
**blank 1**	0.0	0.0	3.7	1.0	4.7
**blank 2**	0.0	0.0	7.8	1.2	9.0
**blank 3**	0.0	0.0	3.6	1.1	4.7
**blank 4**	3.2	0.0	0.0	0.6	3.9
**blank 5**	2.4	0.2	0.0	0.5	3.2
**blank 6**	6.5	1.4	0.7	0.7	9.2
**blank 7**	0.0	3.7	6.2	2.6	12.5
**Mean ± SD**	**1.7 ± 2.3**	**0.8 ± 1.3**	**3.1 ± 2.9**	**1.1 ± 0.7**	**6.7 ± 3.2**

The elevated proportion of PP in the NIR and TAM$_{GF\text{-}filters}$ blanks is striking but the source of contamination remains unclear. For the interpretation of the results the found blanks have no substantial influence. So, contamination was evaluated as an approximately negligible factor.

S2 Supplementary Results and Discussion

Table S5 *Overview of studies investigating vertical MP concentration profiles in fluvial sediments*

Site	Sampling method (analyzed volume)	Sediment texture	Concentration (size range)	Polymer type	Trend of MP concentration along depth	Sampling depth and resolution	Reference
Rhine River (Germany)	buckets of a chain dredging vessel (20–60 g)	clay to coarse pebbles*	$3.43 \pm 0.19 \times 10^3$–$0.89 \pm 0.05 \times 10^3$ MP/kg (11–5000 µm)	PU, CPE, PET, PE, PS, EVA, PP, PA	decrease	7 cm, 42 cm and 111 cm	Mani et al. (2019)
Pearl River estuary (China)	gravity corer (-*)	-*	140–820 MP/kg (<250–5000 µm*)	PP, PE, PET, PS and PC	decrease (overall)<br><br>increase (< 450 µm)	0–54 cm (2 cm steps)	Fan et al. (2019)
Roter Main River (Germany)	freeze core (~50 g)	sand, medium/ coarse gravel, cobbles	0–2.2×10^{-3} MP/kg (500–5000 µm)<br><br>10×10^3–50×10^3 MP/kg (20–500 µm)	PAN, PS, EP, PTFE, PP, PUR and PE (500–5000 µm)<br><br>PP, PVC and PET, PS and PE (20–500 µm)	no trend (overall)<br><br>decrease (20–500 µm)	0–60 cm (10 cm steps)	Frei et al. (2019)
Qiantang River (China)	gravity corer (100 g)	-*	40–550 MP/kg (500–5000 µm)	PE, PS, PET, PS-PP, Cellulose and PP-PE	decrease	0–15 cm (5 cm steps)	Fraser et al. (2020)
Fuhe River (China)	columnar sampler (50 g)	estuary sediments*	5.7 ± 9.6–570.9 ± 280.7 MP/kg (630–5000 µm)	PE, PP, PET, PA and PS	exponential decrease	0–50 cm (5 cm steps)	Zhou et al. (2021)
Qinhuai River (China)	perspex tubes + gravity corers (-*)	silt*	163–563 MP/kg (300–5000 µm)	PE, PP, PMMA and PU	linear increase (overall)<br><br>increase (< 2mm)<br><br>decrease (> 2mm)	0–50 cm (10 cm steps)	Niu et al. (2021)
Yangtze River (China)	push corer (50 g)	-*	207 ± 95–1817 ± 467 MP/kg (<200–>1000 µm*)	PP, PE, PS, PET and PVC	decrease	0–50 cm (10 cm steps)	Bao et al. (2023)
Main River (Germany)	freeze core **(150 g)**	sand or gravel with armourstones at top	21.7 ± 21.4 MP/kg (104–1770 µm)<br><br>30.6 ± 27.8 mg/kg	PE, PP, PS, PVC, PET	no trend (0–30 cm depth)<br><br>decrease (30–60 cm depth)<br><br>increase (60–100 cm depth)	**0–100 cm** (10 cm steps)	Present study

* No detail information provided/found.

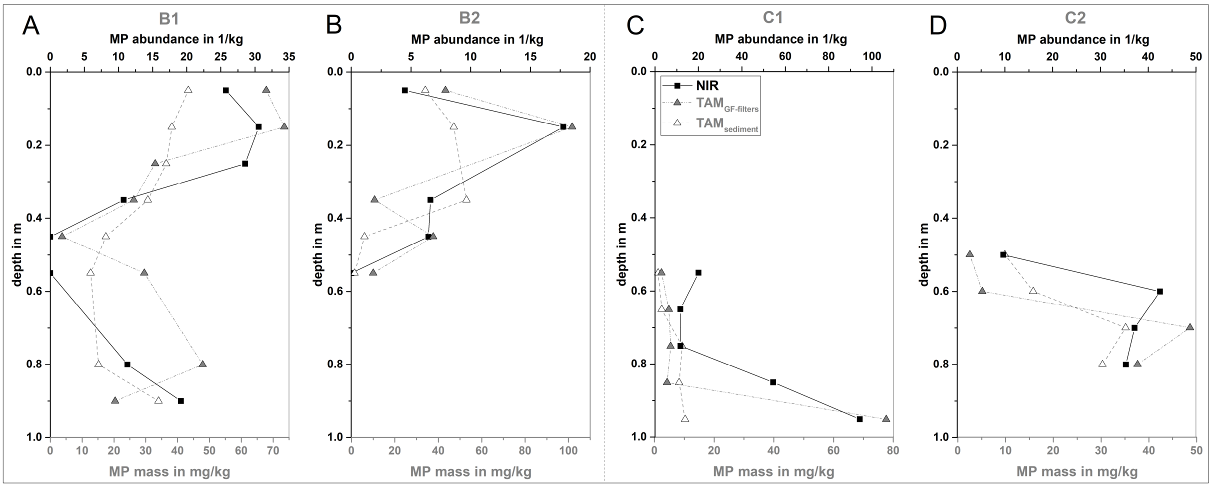

Fig. S3 *Depth-specific MP concentration profiles for each freeze core resulting from the three analytical workflows*

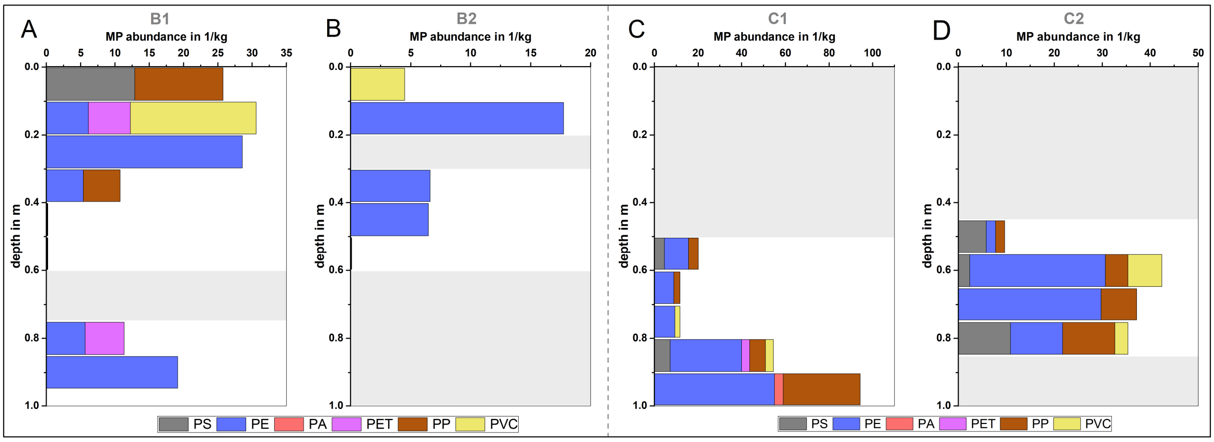

Fig. S4 *Depth-specific MP abundance and polymer type for each freeze core resulting from NIR; in gray-shaded areas not enough fine sediment could be extracted for MP analysis due to the high-volume fraction of armourstones or organic material strongly dominating there*

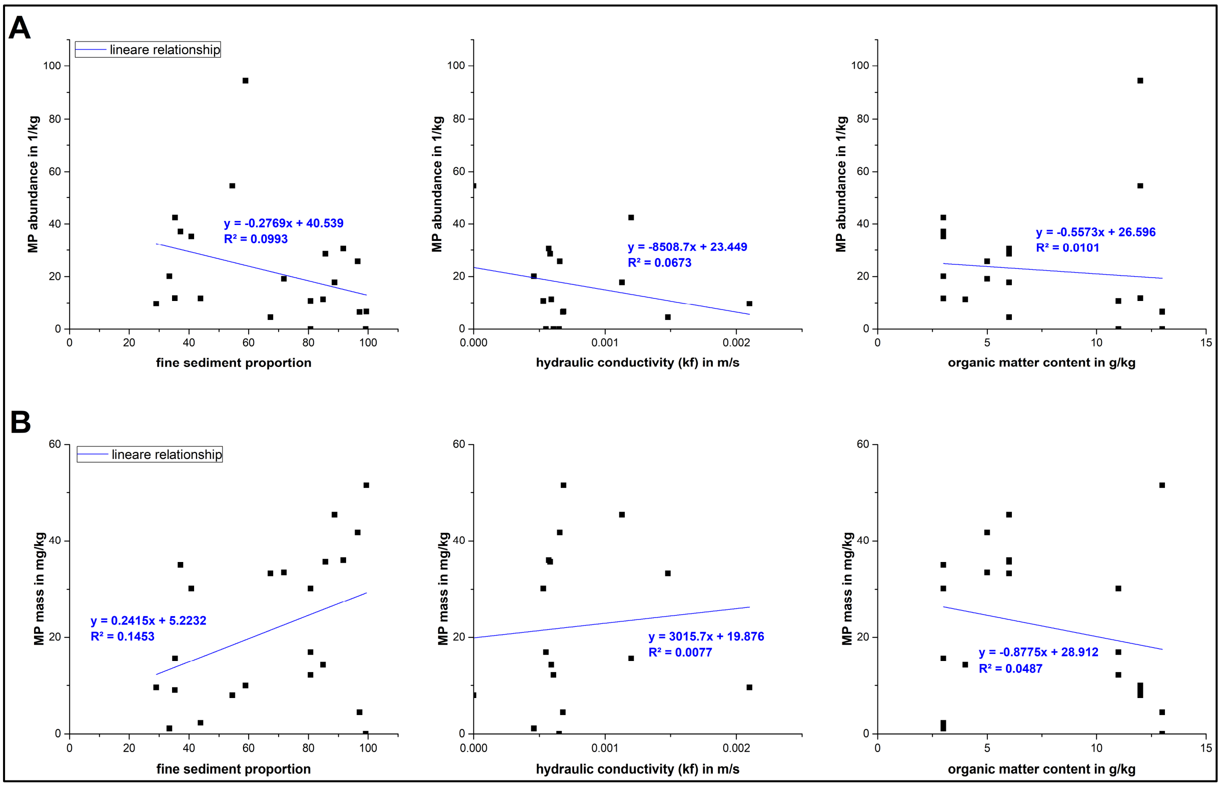

Fig. S5 *Linear relationship between the depth-specific MP abundance (A) or masses (B), and the corresponding proportion of fine sediment or hydraulic conductivity (k_f) or content of organic matter content of the riverbed*

Table S6 *Total sediment dry weight of each freeze core depth-segment sample*

Sediment core	Depth-segment in m	Sediment dry weight in g
**B1**	0.00–0.10	1303.1
	0.10–0.20	925.8
	0.20–0.30	1143.6
	0.30–0.40	1167.5
	0.40–0.50	1481.7
	0.50–0.60	1558.1
	0.75–0.85	1225.8
	0.85–0.95	1417.4
**B2**	0.00–0.10	1647.7
	0.10–0.20	1404.2
	0.30–0.40	836.7
	0.40–0.50	972.6
	0.50–0.60	892.9
**C1**	0.50–0.60	3920.3
	0.60–0.70	2430.2
	0.70–0.80	2693.8
	0.80–0.90	1875.2
	0.90–1.00	1946.6
**C2**	0.45–0.55	4203.5
	0.55–0.65	3324.8
	0.65–0.75	2509.4
	0.75–0.85	2673.9

References

Bao K, Jiang H, Su P, Lu P, Yan Z (2023) Vertical Profiles of Microplastics in the Hyporheic Zone Sediment: A Case Study in the Yangtze River, Nanjing Section. Sustainability 15(10):7895. doi: 10.3390/su15107895

DIN 18128 (2002) Soil - Investigation and testing - Determination of ignition loss. DIN 18128:2002-12 Beuth Verlag GmbH. doi: 10.31030/9287613

Fan Y, Zheng K, Zhu Z, Chen G, Peng X (2019) Distribution, sedimentary record, and persistence of microplastics in the Pearl River catchment, China. Environ. Pollut. 251:862–870. doi: 10.1016/j.envpol.2019.05.056

Fraser MA, Chen L, Ashar M, Huang W, Zeng J, Zhang C, Zhang D (2020) Occurrence and distribution of microplastics and polychlorinated biphenyls in sediments from the Qiantang River and Hangzhou Bay, China. Ecotox. Environ. Safe. 196:110536. doi: 10.1016/j.ecoenv.2020.110536

Frei S, Piehl S, Gilfedder BS, Löder MGJ, Krutzke J, Wilhelm L, Laforsch C (2019) Occurence of microplastics in the hyporheic zone of rivers. Sci. Rep. 9(15256). doi: 10.1038/s41598-019-51741-5

Mani T, Primpke S, Lorenz C, Gerdts G, Burkhardt-Holm P (2019) Microplastic Pollution in Benthic Midstream Sediments of the Rhine River. Environ. Sci. Technol. 53(10):6053–6062. doi: 10.1021/acs.est.9b01363

Munz M, Kreiß J, Krüger L, Schmidt LK, Bochow M, Bednarz M, Bannick CG, Oswald SE (2023) Application of High-Resolution Near-Infrared Imaging Spectroscopy to Detect Microplastic Particles in Different Environmental Compartments. Water Air Soil Pollut. 234(5):286. doi: 10.1007/s11270-023-06245-4

Niu L, Li Y, Li Y, Hu Q, Wang C, Hu J, Zhang W, Wang L, Zhang C, Zhang H (2021) New insights into the vertical distribution and microbial degradation of microplastics in urban river sediments. Water Res. 188(116449). doi: 10.1016/j.watres.2020.116449

Schmidt LK, Bochow M, Imhof HK, Oswald SE (2018) Multi-temporal surveys for microplastic particles enabled by a novel and fast application of SWIR imaging spectroscopy – Study of an urban watercourse traversing the city of Berlin, Germany. Environ. Pollut. 239:579–589. doi: 10.1016/j.envpol.2018.03.097

Stocker ZS, Williams DD (1972) Freezing core method for describing the vertical distribution of sediments in a streambed. Limnol. Oceanogr. 17:1. doi: 10.4319/lo.1972.17.1.0136

Strasser D, Lensing H-J, Nuber T, Richter D, Frank S, Goeppert N, Goldscheider N (2015) Improved geohydraulic characterization of river bed sediments based on freeze-core sampling – Development and evaluation of a new measurement approach. J. Hydrol. 527:133–141. doi: 10.1016/j.jhydrol.2015.04.074

Straßer D, Lensing H-J, Richter D, Frank S, Goldscheider N (2014) Die Gefrierkernmethode - Weiterentwicklung des Erkundungsverfahrens zur geohydraulischen Charakterisierung von Sohlsedimenten. In: Bundesanstalt für Wasserbau (ed) Aktuelle Themen der Geotechnik. Herle, Ivo, Karlsruhe, pp 123–136

Zhou Z, Zhang P, Zhang G, Wang S, Cai Y, Wang H (2021) Vertical microplastic distribution in sediments of Fuhe River estuary to Baiyangdian Wetland in Northern China. Chemosphere 280:130800. doi: 10.1016/j.chemosphere.2021.130800
